# Noise Exposure Alters Glutamatergic and GABAergic Synaptic Connectivity in the Hippocampus and Its Relevance to Tinnitus

**DOI:** 10.1155/2021/8833087

**Published:** 2021-01-14

**Authors:** Liqin Zhang, Calvin Wu, David T. Martel, Michael West, Michael A. Sutton, Susan E. Shore

**Affiliations:** ^1^Kresge Hearing Research Institute, Department of Otolaryngology, University of Michigan, Ann Arbor, Michigan, USA; ^2^Molecular & Behavioral Neuroscience Institute, University of Michigan, Ann Arbor, Michigan, USA; ^3^Department of Otolaryngology, Peking Union Medical College Hospital, Beijing, China; ^4^Xiangya Medical School, Central South University, Changsha, Hunan, China; ^5^Department of Biomedical Engineering, University of Michigan, Ann Arbor, Michigan, USA; ^6^Department of Molecular and Integrative Physiology, University of Michigan, Ann Arbor, Michigan, USA

## Abstract

Accumulating evidence implicates a role for brain structures outside the ascending auditory pathway in tinnitus, the phantom perception of sound. In addition to other factors such as age-dependent hearing loss, high-level sound exposure is a prominent cause of tinnitus. Here, we examined how noise exposure altered the distribution of excitatory and inhibitory synaptic inputs in the guinea pig hippocampus and determined whether these changes were associated with tinnitus. In experiment one, guinea pigs were overexposed to unilateral narrow-band noise (98 dB SPL, 2 h). Two weeks later, the density of excitatory (VGLUT-1/2) and inhibitory (VGAT) synaptic terminals in CA1, CA3, and dentate gyrus hippocampal subregions was assessed by immunohistochemistry. Overall, VGLUT-1 density primarily increased, while VGAT density decreased significantly in many regions. Then, to assess whether the noise-induced alterations were persistent and related to tinnitus, experiment two utilized a noise-exposure paradigm shown to induce tinnitus and assessed tinnitus development which was assessed using gap-prepulse inhibition of the acoustic startle (GPIAS). Twelve weeks after sound overexposure, changes in excitatory synaptic terminal density had largely recovered regardless of tinnitus status, but the recovery of GABAergic terminal density was dramatically different in animals expressing tinnitus relative to animals resistant to tinnitus. In resistant animals, inhibitory synapse density recovered to preexposure levels, but in animals expressing tinnitus, inhibitory synapse density remained chronically diminished. Taken together, our results suggest that noise exposure induces striking changes in the balance of excitatory and inhibitory synaptic inputs throughout the hippocampus and reveal a potential role for rebounding inhibition in the hippocampus as a protective factor leading to tinnitus resilience.

## 1. Introduction

Tinnitus is a phantom perception of sound (e.g., ringing in the ears) in the absence of corresponding external auditory stimuli, which affects millions of people worldwide [1, 2]. Tinnitus is debilitating for 1-2% of the general population who seek medical help [3, 4] and is comorbid with anxiety [5], depression [6, 7], and emotional distress [8], all of which significantly affect quality of life. The most common factor associated with tinnitus is noise overexposure [9]. Therefore, noise exposure is widely used as a method to induce tinnitus in animal models [10–14].

Previous studies indicate that most cases of chronic tinnitus develop as a consequence of neuroplasticity in central auditory pathways and other brain regions following reduced auditory input [15]. The hippocampus plays essential role in learning, memory, and mood [16, 17], and hippocampal neuroplasticity can be impacted by early auditory training [18]. Previous studies implicate the hippocampus in tinnitus [19–22]. Acute, high-intensity noise induces rapid Arc protein expression in the rat hippocampus [23], alters the responses of hippocampal place cells [21], and impairs hippocampal-dependent learning [24]. In humans, it has been demonstrated by resting-state fMRI that tinnitus loudness is positively correlated with bilateral hippocampal activity [22], while BOLD fMRI has revealed a decline in functional connectivity between lower auditory brainstem regions and hippocampus in tinnitus patients [25]. Collectively, these findings suggest a potential role for the hippocampus in the altered auditory processing associated with tinnitus.

Neural information processing relies on the proper balance of excitation and inhibition. In the hippocampus, a proposed basic route of information processing occurs via excitatory glutamatergic projections forming the so-called “trisynaptic circuit” [26, 27], characterized by feed-forward excitatory projections via the perforant path from entorhinal cortex (EC) to dentate gyrus (DG), the mossy fiber pathway to hippocampal area CA3, and Schaffer collateral projections to area CA1 [28].

The information flow in the “trisynaptic circuit” is mediated via neurotransmission, in which vesicular transporters play essential roles. A vesicular transporter is a membrane protein that regulates or facilitates the movement of specific molecules across a vesicle's membrane, thus governing the concentration of molecules within a vesicle. Vesicular glutamate transporters form a family of proteins responsible for the transport of glutamate into vesicles, thus playing an indispensable role in glutamatergic transmission. VGLUT-1 is the predominant isoform in the hippocampus, which critically affects the efficacy of hippocampal glutamatergic synaptic transmission [29, 30]. VGLUT-2 is the major isoform in thalamocortical sensory projections and is localized to the granule cell layer of the DG [31]. VGLUT-2 is pivotal to the proper development of mature pyramidal neuronal architecture and plasticity in the hippocampus [32]. Immunohistochemistry and in situ hybridization of VGAT revealed that the transporter is expressed in the GABAergic and glycinergic neurons of neocortex, hippocampus, cerebellum, striatum, septal nuclei, and reticular nucleus of the thalamus [33, 34]. Inhibition of the principal neurons in the hippocampus is performed mainly by a heterogeneous group of GABAergic interneurons, which regulate the overall level of excitability in the network and contribute to hippocampal oscillations [35]. Dysfunction of GABAergic inhibition is related to several neurological diseases including epilepsy [36–38], schizophrenia [39, 40], and autism [41, 42]. Furthermore, noise-exposed rats exhibit reduced GABA levels and upregulation of glutamic acid levels in the hippocampus and performed worse on Morris Water Maze tests [43]. These findings raise the question of whether hippocampal glutamatergic and GABAergic innervation is affected by noise exposure, whether these changes are persistent, and whether they are associated with tinnitus.

Here, we examined immunohistochemical labeling of VGLUT-1, VGLUT-2, and the vesicular GABA transporter (VGAT) to investigate glutamatergic and GABAergic innervation in the hippocampus after noise exposure and further explored the relevance of these changes to tinnitus. Two weeks after a unilateral noise exposure, we found primarily opposite changes in excitatory and inhibitory synapse density throughout the hippocampus—VGLUT-1 density largely increased, while VGAT density exhibited robust decreases in numerous hippocampal subregions, including the DG and areas CA3 and CA1. All these changes were evident bilaterally. To investigate whether these changes were persistent and potentially relevant to tinnitus, we designed a second experiment using animals exposed to the same noise band twice four weeks apart, a paradigm that induces tinnitus in a subset of animals [11, 44]. Twelve weeks after the initial noise exposure, VGLUT-1 and VGLUT-2 density recovered regardless of tinnitus phenotype, but VGAT density recovery was markedly different depending on whether the animals exhibited tinnitus: exposed animals that were resistant to tinnitus showed similar or even higher levels of VGAT labeling compared to controls in every hippocampal subregion examined, while animals that developed tinnitus exhibited persistent reduction in VGAT density in the DG and areas CA3 and CA1. Collectively, our results identify a novel association between remodeling of GABAergic innervation in the hippocampus and the pathophysiology of noise-induced tinnitus.

## 2. Materials and Methods

### 2.1. Animals

The same animals were used as in a previously published work from our lab [45]. Pigmented guinea pigs of either sex (*n* = 19) were obtained from Elm Hill Labs at 2-3 weeks of age. Animals were housed two per cage in a temperature and humidity-controlled environment and had free access to water and food. All animal procedures were performed in accordance with protocols established by the National Institutes of Health and approved by the University Committee on Use and Care of Animals at the University of Michigan.

### 2.2. Experimental Design

**Figure 1:**
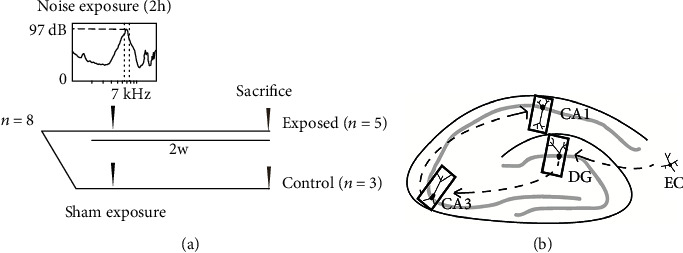
Experimental procedures of two-week postnoise-exposure animals. (a) Timeline of the experimental procedures for the two-week postexposure group. Eight animals were grouped into sham-exposed controls (*n* = 3) and noise-exposed animals (*n* = 5). Animals were exposed to a 7 kHz-centered noise band at 97 dB SPL for 2 hours unilaterally (left). ABR measurements were performed before, immediately after, and 2 weeks after noise exposure. Two weeks after noise exposure, animals were sacrificed, and brain tissue was collected. (b) Schematic diagram of hippocampal circuit. Arrowheads in dashed line show information flow in the hippocampal trisynaptic circuit. Black rectangles point out where images in the dentate gyrus (DG) and areas CA3 and CA1 were taken for immunohistochemistry. EC: entorhinal cortex.

The first experiment was designed to investigate the effects of noise exposure on the hippocampus (Figure 1(a)). Animals were divided into noise-exposure group (*n* = 5) and Sham controls (*n* = 3). Animals were noise-exposed/sham-exposed once. Auditory brainstem responses (ABRs) were recorded in response to tone bursts before, immediately after, and two weeks after the noise exposure to assess shifts in hearing thresholds. Two weeks following the noise exposure, animals were sacrificed, and brains were collected as described below in *brain collection and tissue preparation.*

**Figure 2:**
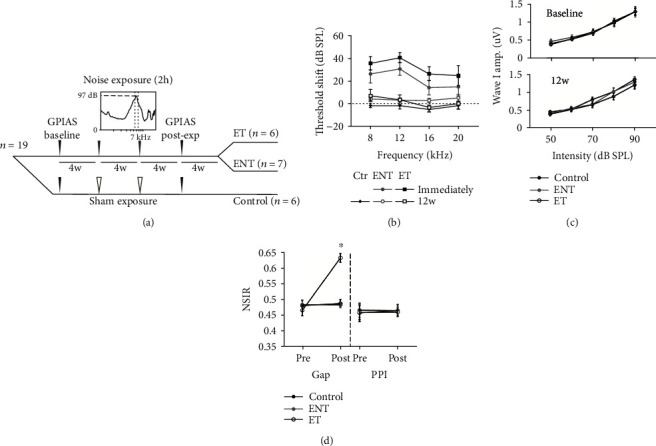
Repeated noise exposure induces tinnitus in a subset of animals. (a) Timeline of the experimental procedures of the chronically exposed group. Nineteen animals were grouped into sham controls (*n* = 6) and noise-exposed animals (*n* = 13). GPIAS was used for tinnitus assessment, and the baseline of GPIAS was acquired for four weeks before noise exposure. Animals were noise-exposed to the same noise band/sham for two hours twice in sessions conducted four weeks apart, and then assessed for tinnitus with GPIAS eight weeks following the first noise exposure. ABR measurements were performed before and after each noise exposure and GPIAS. Noise-exposed animals were divided into two further groups according to GPIAS assessment results: noise exposed animals that exhibit no behavioral evidence signs of tinnitus (ENT, *n* = 7) and exposed animals that exhibit behavioral evidence of tinnitus (ET, *n* = 6). (b) Mean (±SEM) ABR thresholds of animals with tinnitus (ET) and without tinnitus (ENT). ABR thresholds on the ipsilateral side were elevated immediately following noise exposure in both groups, but recovered to baseline levels at 8, 12, 16, and 20 kHz 12 weeks after the first noise exposure. (c) Mean (±SEM) ABR wave I amplitude-intensity functions for ENT and ET animals pre- (baseline) and postnoise exposure (12w) were not significantly different, suggesting no underlying cochlear synaptopathy in both ENT and ET animals after the noise exposure. (d) Mean (±SEM) normalized startle inhibition ratio (NSIR) was the ratio of the startle amplitudes for the gap (or prepulse inhibition, PPI) trials and those for the no-gap trials. NSIR for gap trials was significantly higher postexposure (post) relative to baseline levels (pre) for ET animals, but not for ENT or control animals. All animals exhibited stable responses to PPI trials both pre- and postnoise exposure. ^∗^*p* ≤ 0.05. This figure is reproduced with permission of authors in Zhang et al., 2019.

The second experiment was conducted to further investigate whether the changes seen two-week postnoise exposure persisted and evaluate its relevance to tinnitus (Figure 2(a)). Animals (*n* = 19) were exposed to the same noise band (*n* = 13)/sham (*n* = 6) twice four weeks apart and assessed for tinnitus development using gap-prepulse-inhibition of acoustic startle reflex (GPIAS). Baseline data were collected 4 weeks prior to the first noise exposure, and GPIAS experiments were performed twice a week for 4 weeks. The postexposure data was collected 8 weeks after the first noise exposure, and the frequency and duration of the experiment were the same as at baseline. All GPIAS experimental data were collected within twelve weeks of the first noise exposure. The noise-exposed animals were further divided into exposed-no-tinnitus (ENT, *n* = 6) group and exposed-tinnitus (ET, *n* = 7) group according to the GPIAS results. ABRs were measured before and immediately after each noise exposure and by the end of GPIAS tests. Animals were sacrificed by the end of GPIAS tests. Immunohistochemical staining was performed with brain sections from both experiments to evaluate the changes of glutamatergic and GABAergic neurotransmission in hippocampus and their correlation with tinnitus.

### 2.3. Noise Exposure and ABR Measurement

Noise exposure and ABR were conducted in a double-wall soundproof booth as previously described [11, 44]. Guinea pigs were anesthetized with ketamine/xylazine (40 mg/kg ketamine; 10 mg/kg xylazine), and were then unilaterally exposed to a 7 kHz centered noise band at 97 dB SPL for 2 hours via a closed speaker system inserted in the left ear canal (Figure 1(a)). Auditory brainstem responses(ABRs) were recorded in response to tone bursts (0.5 ms cosine squared gating, 5 ms duration) at 8, 12, 16, and 20 kHz, before and after each noise exposure to assess shifts in hearing thresholds and wave I amplitude-intensity functions as previously described [45]. ABRs were also assessed two weeks after the noise exposure to determine hearing threshold recovery.

### 2.4. Tinnitus Assessment

Gap-prepulse inhibition of acoustic startle (GPIAS) was used for tinnitus assessment and performed as previously described [11, 44]. A constant background carrier (band limited at 8-10, 12–14, 16–18, and 20-30 kHz) was presented at 65 dB SPL. Because the guinea-pig pinna reflexes to startle pulses have been shown to be less variable than whole body startles [46], we quantified pinna reflex startle responses evoked by broadband noise pulses (20 ms) at 95 dB SPL. Startle reflexes were inhibited by a silent gap (50 ms) or prepulse noise (75 dB SPL) 100 ms before the startle pulse. To quantify the pinna reflex amplitude, pinna tips of animals were marked with nontoxic, water-soluble green paint, manually applied by trained investigators. Green pixels were identified using a custom-written *k*-nearest neighbor classifier algorithm (Mathworks MATLAB). Frames where green points constituted less than 0.01% of pixels were excluded, as this indicated the animal's ears were not located in the frame. Pinna locations were identified by clustering green pixels and computing the centroids of a two-dimensional Gaussian mixture model. The Euclidean distance between (Xear(*t*), Year (*t*)) points was computed over the trial duration. Startle amplitudes were computed by fitting the Euclidean distance to a Gaussian-windowed sine-wave cycle and computed as the resultant amplitude parameter.

(1) $$\begin{array}{c} \text{SR}=\frac{\text{mean startle amplitude for gap}\left(\text{prepulse}\right)\text{ trials}}{\text{mean startle amplitude for nogap}\left(\text{prepulse}\right)\text{ trials}},\\ \text{Tinnitus index}=\frac{x_{\text{post}}-\mu_{\text{pre}}}{\sigma_{\text{pre}}}. \end{array}$$

A normalized startle ratio (SR) was defined as the ratio of the mean startle amplitude for the gap (or prepulse noise) trials and those for the no-gap trials. Tinnitus index, as shown by the equation above, was used to quantify the difference between postexposure and preexposure. *x*_post_ is the mean of postexposure SR value. *μ*_pre_ and *σ*_pre_ are the mean and standard deviation (SD) of preexposure SR value, which was the behavioral baseline. By twelve weeks after the initial noise exposure, GPIAS data collection was completed and SR values for each animal were calculated. If an animal showed significantly greater normalized gap-startle response postexposure compared to preexposure, and not significantly increased prepulse noise startle responses, then an animal was considered to have tinnitus at that frequency band.

In a separate testing session, an equivalent number of noise-prepulses (75 dB; 50 ms; 5 ms rise-fall times), with frequencies used in gap-prepulse testing, were also presented prior to the startle pulse as an assessment of hearing function. Postnoise exposure, no animals demonstrated significant increases in noise prepulse inhibition at any testing frequency, compared to prenoise exposure.

Because threshold shifts or deficits in suprathreshold hearing can affect GPIAS outcome [47], to ensure the deficits in gap detection resulted from tinnitus instead of hearing loss, we used a titrated noise exposure that only causes temporary threshold shift and no observable suprathreshold deficits [11, 44]. ABR wave I amplitude-intensity functions were used to identify suprathreshold hearing deficits in animals [48]. Prepulse startle amplitudes did not change significantly over the course of testing.

### 2.5. Tissue Preparation and Immunohistochemistry

The procedures of brain collection, tissue preparation, and immunohistochemistry were as described before [45]. Hippocampus was sectioned into five series of coronal sections (30 *μ*m) with a cryostat (Leica, CM 3050S), mounted on glass slides, air dried for 24 hours, and stored at -20°. Before staining, slides were allowed to thaw at room temperature for one hour after removal from -20°C. All subsequent procedures were performed at room temperature. Brain sections were rehydrated in PBS for 30 min to optimize morphological details. Subsequently, sections were incubated in blocking solution consisting of 1% normal goat serum (Jackson ImmunoResearch Labs, Cat# 005-000-121, RRID: AB_2336990) and 0.1% Triton-X 100 (MP Biomedicals, Cat# 807423) in PBS for 30 min, to limit nonspecific binding. Sections were then incubated with primary antibody, rabbit anti-VGLUT-1 antibody (Synaptic Systems, Cat# 135 303, RRID: AB_887875), 1 : 1000 diluted in blocking solution, or rabbit anti-VGLUT-2 antibody (Synaptic Systems, Cat# 135 403, RRID: AB_887883), 1 : 500 diluted in blocking solution, or rabbit anti-VGAT antibody (Synaptic Systems, Cat# 131 003, RRID: AB_887869), and 1 : 200 diluted in blocking solution for 24 hours. After thorough rinsing (10 min^∗^3 times) in PBS to remove unbound primary antibody, all sections were incubated with secondary antibody (Alexa Fluor 555-conjugated goat anti-rabbit, Molecular Probes Cat# A-21429, RRID: AB_141761) diluted 1 : 500 in blocking solution for two hours. DAPI (Thermo Fisher Scientific, Cat# D1306, RRID: AB_2629482) 1 : 1000 diluted in blocking solution was applied together with the secondary antibody for counterstain. After the incubation, another thorough rinsing (10 min^∗^3 times) was performed to remove excess secondary antibody. Slides were mounted with Fluoromount-G (Southern Biotech, Cat# 0100-01). To ensure specificity of the secondary antibody, negative controls were done in sections only treated with secondary (and not primary) antibody. All matched groups were processed in parallel.

### 2.6. Image Processing and Quantification

Image processing was performed as previously described [45, 49]. The control and exposed animal images were acquired, processed, and analyzed using identical methods. The experimenters were blind with respect to treatment at the time of image quantification. The ROIs were selected based on anatomical landmarks referring to the Allen Brain Atlas and relevant staining characteristics of DAPI, VGAT, VGLUT-1, and VGLUT-2 to ensure that ROI's demarcate analogous areas of hippocampal subregions. Images for VGLUT-1 and VGAT were taken from Dentate Gyrus (DG), area CA3, and area CA1 (Figure 1(b)). VGLUT-2 staining was only quantified for the granule cell layer of the DG. For VGLUT-1 and VGAT staining quantification, the molecular layer of the DG was subdivided into three equally sized regions—proximal, middle, and distal regions. The proximal part is adjacent to the granule cell layer. In CA1, the stratum radiatum was subdivided into two equally sized regions—proximal and distal regions. Quantification of puncta density was performed with Image J (version 1.50i, National Institutes of Health, USA, RRID: SCR_003070) with a custom macro. First, RGB images were converted into single channels, and only the red channel corresponding to the Alexa Fluor 555 signal was used for subsequent processing. Then, the contrast was enhanced, and background was subtracted with consistent parameters. Subsequently, an auto threshold was applied followed by a watershed paradigm which separated overlapping puncta. Puncta density was yielded by dividing puncta counts by respective image area. All the above steps were run automatically in Image J with a set macro, and all parameters for a certain staining were consistent.

### 2.7. Statistics

Statistical analysis was done with MATLAB (The MathWorks, RRID: SCR_001622). One-sample student test, two-sample student test, and two-way ANOVA followed by Tukey-Kramer post hoc correction for multiple comparisons were used (*p* ≤ 0.05).

## 3. Results

### 3.1. Hippocampal VGLUT and VGAT Density Were Altered Two Weeks following Noise Exposure

To determine whether altered glutamatergic and GABAergic innervation in the hippocampus accompanies noise exposure, we used immunohistohemical detection of VGLUT-1, VGLUT-2, and VGAT to identify glutamatergic and GABAergic terminals in hippocampal subregions. Immediately after noise exposure, animals exhibited elevated ABR thresholds on the ipsilateral side at 8, 12, 16, and 20 kHz and recovered to baseline levels at 8 and 20 kHz within one week [45]. Control animals and contralateral sides of exposed animals did not exhibit any changes in ABR thresholds using this noise exposure paradigm in previous studies [11, 44]. ABR wave I amplitude-intensity functions did not show any differences from baseline levels in exposed animals two weeks following the noise exposure, suggesting that the exposed animals did not have any observable suprathreshold hearing impairments.

### 3.2. VGLUT-1 Labeling in the Hippocampus Was Increased Two Weeks after Noise Exposure

**Figure 3:**
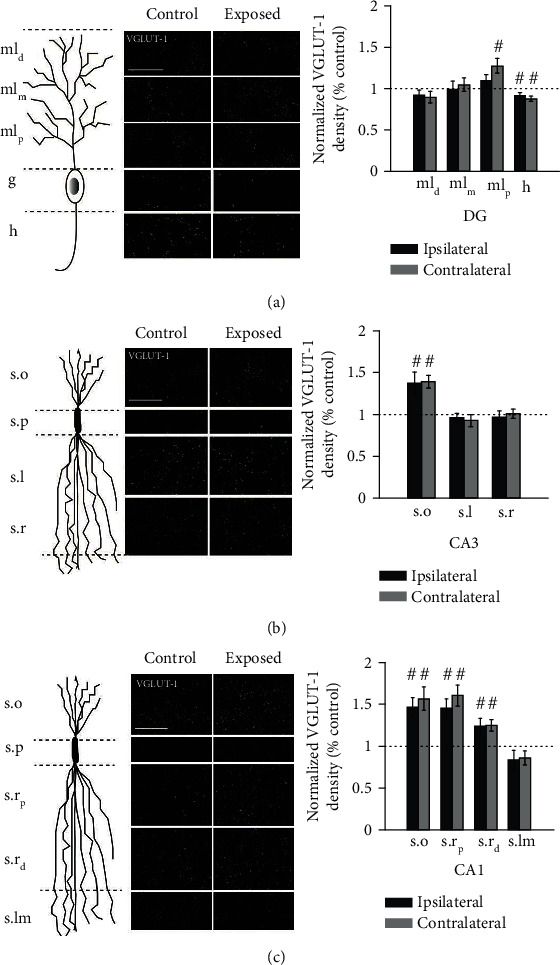
VGLUT-1 puncta density primarily increases in dentate gyrus (DG), area CA3, and area CA1 two weeks after noise exposure. Schematic granule cell in DG (a), pyramidal neuron in CA3 (b), and pyramidal neuron in CA1 (c), depicting organization of inputs corresponding to the layers on the right, which are images of VGLUT-1 labeling in CA1 at 400x magnification. Scale bar, 50 *μ*m. Mean (±SEM) normalized VGLUT-1 puncta density (per 10^4^ *μ*m^2^; normalized to respective control) in the dentate gyrus (DG) (a), area CA3 (b), and area CA1 (c) two weeks following noise exposure are shown on respective right panels. ^#^When compared to the controls (dashed line), *p* ≤ 0.05. ml_d_: distal region of molecular layer; ml_m_: middle region of molecular layer; ml_p_: proximal region of molecular layer; h: hilus; s.o: stratum oriens; s.l: stratum lucidum; s.r: stratum radiatum; s.r_p_: proximal region of stratum radiatum; s.r_d_: distal region of stratum radiatum; s.lm: stratum lacunosum-moleculare.

VGLUT-1 is the predominant isoform of VGLUT in the hippocampus. VGLUT-1-positive puncta were detected throughout the hippocampus except for the pyramidal and granule cell layers. Although the noise exposure was unilateral, we found changes in VGLUT-1 expression on both sides of the hippocampus two-week postexposure (Figure 3). In the DG, the proximal molecular layer exhibited increases in VGLUT-1 puncta density after noise exposure, but only the changes on the contralateral side reached statistical significance. The hilus showed significant reductions in VGLUT-1 density on both sides. The distal and middle regions of the molecular layer did not show any significant changes compared to controls.

In hippocampal area CA3, only stratum oriens showed significantly increased VGLUT-1 density in exposed animals on both sides (ipsilateral(I): *p* ≤ 0.01; contralateral(C): *p* ≤ 0.001). No significant change was seen in stratum lucidum and radiatum.

In area CA1, most layers, including stratum oriens (s.o), proximal (s.r_p_), and distal (s.r_d_) regions of stratum radiatum showed significant increases in VGLUT-1 density on both sides(s.o, I: *p* ≤ 0.001; C: *p* ≤ 0.001; s.r_p_, I: *p* ≤ 0.001; C: *p* ≤ 0.001; s.r_d_, I: *p* ≤ 0.01; C: *p* ≤ 0.01). Stratum lacunosum-moleculare (s.lm) exhibited a slight reduction in VGLUT-1 expression, but the difference did not reach statistical significance. Most prominent changes were observed on both sides of areas CA3 and CA1 despite the unilateral nature of the noise exposure. Together, these results demonstrate layer-specific changes in glutamatergic inputs to the hippocampus two weeks following noise exposure.

### 3.3. VGLUT-2 Labeling in the Hippocampus Was Decreased Two Weeks after Noise Exposure

**Figure 4:**
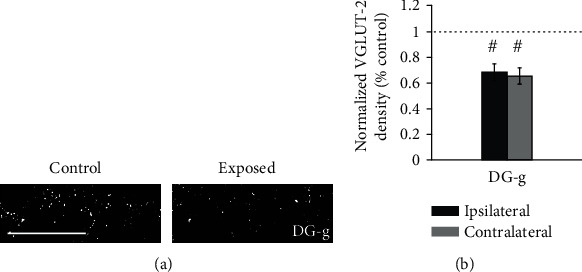
VGLUT-2 puncta density decreases in the granule cell layer of the dentate gyrus two weeks following noise exposure. (a) Representative images of VGLUT-2 labeling in the granule cell layer of the DG (DG-g) at 400x magnification. Scale bar, 50 *μ*m. (b) Mean (±SEM) VGLUT-2 puncta density (per 10^4^ *μ*m^2^; normalized to respective control) in the granule cell layer. ^#^When compared to the controls (dashed line), *p* ≤ 0.05.

VGLUT-2-positive puncta mainly localize to the granule cell layer of the dentate gyrus. Compared to controls, exposed animals exhibited significantly lower levels of VGLUT-2 density on both ipsilateral and contralateral sides in the granule cell layer (I: *p* ≤ 0.001; C: *p* ≤ 0.001) (Figure 4). Again, while the noise exposure was unilateral, robust changes in VGLUT-2 density were observed in hippocampi on both sides of the brain.

### 3.4. VGAT Labeling in the Hippocampus Was Decreased Two Weeks after Noise Exposure

**Figure 5:**
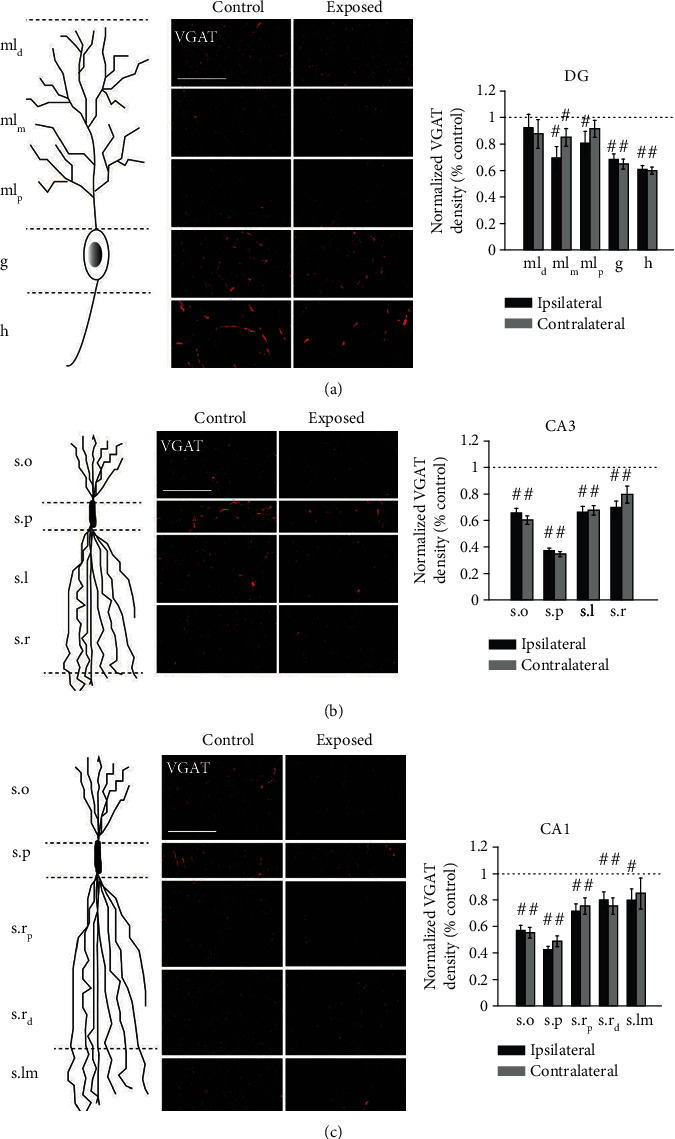
VGAT puncta density decreases in dentate gyrus (DG), area CA3, and area CA1 two weeks following noise exposure. Schematic granule cell in DG (a), pyramidal neuron in CA3 (b), and pyramidal neuron in CA1 (c), depicting organization of inputs corresponding to the layers on the right, which are images of VGAT labeling in CA1 at 400x magnification. Scale bar, 50 *μ*m. Mean (±SEM) VGAT puncta density (per 10^4^ *μ*m^2^; normalized to respective control) in the dentate gyrus (DG) (a), area CA3 (b), and area CA1 (c) two weeks following noise exposure is shown on respective right panels. ^#^When compared to the controls (dashed line), *p* ≤ 0.05. ml_d_: distal region of molecular layer; ml_m_: middle region of molecular layer; ml_p_: proximal region of molecular layer; g: granule cell layer; h: hilus; s.o: stratum oriens; s.p: stratum pyramidale; s.l: stratum lucidum; s.r: stratum radiatum; s.r_p_: proximal region of stratum radiatum; s.r_d_: distal region of stratum radiatum; s.lm: stratum lacunosum-moleculare.

VGAT-positive puncta were detected throughout the hippocampus (Table 1). Except for the distal region of the molecular layer, all other DG layers examined, including the middle region of molecular layer (ml_m_), granule cell layer (g), and hilus (h), exhibited significant bilateral reductions in VGAT density (Figure 5). The proximal region of the molecular layer was significantly decreased only on the ipsilateral side.

Noise-exposed animals demonstrated significantly lower VGAT counts in all four layers of area CA3 compared to controls. Stratum pyramidale (s.p) exhibited the most striking decrease. The other three layers—stratum oriens, lucidum (s.l), and radiatum (s.r)—exhibited moderate, yet significant, reductions in VGAT density.

As observed in CA3, the most striking reduction of VGAT density in area CA1 occurred in stratum pyramidale. Stratum oriens and proximal and distal regions of stratum radiatum all showed significant reductions of VGAT labeling on both sides. Stratum lacunosum-moleculare decreased on both sides, but only reached statistical significance for the ipsilateral side. There were no significant differences in VGAT density between ipsilateral and contralateral sides in exposed animals for all the subregions examined. These results reveal pronounced changes in GABAergic innervation of the hippocampus induced by noise trauma.

### 3.5. Chronic Effects of Noise Exposure and Tinnitus

Given the changes in hippocampal glutamatergic and GABAergic inputs two weeks following noise exposure, we next investigated the persistence of these changes and their association with tinnitus. We thus induced tinnitus in a subset of animals by exposing them to the same noise twice but four weeks apart (Figure 2). Tinnitus was assessed with GPIAS framework as previously described [11]. As the utilized noise exposure paradigm induces tinnitus in roughly half of experimental animals in previous studies [11], chronically exposed animals were divided into three groups: sham exposed controls (*n* = 6), noise exposed animals that exhibit no behavioral evidence of tinnitus (ENT, *n* = 7), and noise exposed animals that exhibited behavioral evidence of tinnitus (ET, *n* = 6).

While GPIAS is a widely used behavioral index of tinnitus, there is currently debate over what aspect of tinnitus alters auditory processing during the gap [50]. To ensure behavioral evidence of tinnitus via GPIAS was not secondary to hearing deficits induced by the noise exposure paradigm, several measures have been taken. Following the dual noise exposure, ipsilateral ABR thresholds of noise-exposed animals (*n* = 13) recovered to baseline levels within 2 weeks and remained recovered at 12-week postexposure [45]. Furthermore, ET and ENT animals exhibited similar ABR wave I amplitude-intensity functions pre- and postnoise exposure [45], suggesting that neither ENT nor ET animals had any supratheshold hearing deficits. The prepulse inhibition (PPI) ratios of the noise-exposed animals remained constant [45], indicating that the animals' attenuated responses to gap trials were not because of hearing impairment. The startle amplitude for no-gap/no-prepulse condition, i.e., the baseline startle reflexivity, remained unaltered postexposure in control, ENT, and ET animals. Given that ABR thresholds recovered to baseline levels 12-week postexposure and that ABR wave I amplitude-intensity functions were the same in ENT and ET animals, the differences observed in gap-startle ratios do not arise due to hearing differences.

### 3.6. VGLUT-1 and VGLUT-2 Labeling Recovered towards Normal Regardless of Tinnitus Expression

Two-way ANOVA with two factors “tinnitus status” and “side” reveals nearly identical noise-induced changes in VGLUT-1 and VGLUT-2 expression in most layers on both sides of the hippocampus for the noise exposure group. Although the “side” effect in the proximal region of stratum radiatum is significant, the interaction of “side” and “tinnitus status” is not significant. VGLUT-1 puncta density comparison with two-way ANOVA is shown in Table 2.

**Figure 6:**
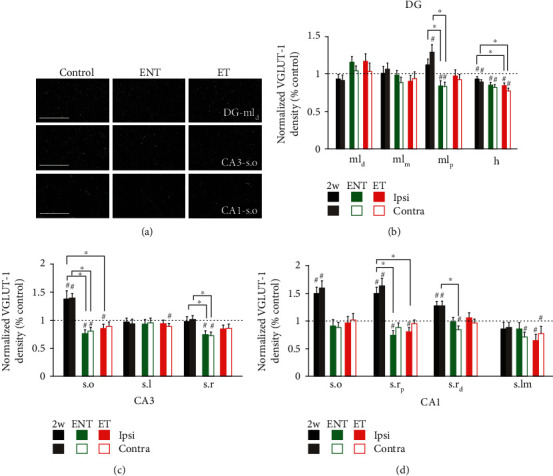
Tinnitus and no-tinnitus animals exhibit recovery of VGLUT-1 labeling by 12-week postnoise exposure in the dentate gyrus (DG), area CA3 and CA1. (a) Representative images of VGLUT-1 labeling from distal molecular layer of the DG (DG-ml_d_), stratum oriens of CA3 (CA3-s.o), and stratum oriens of CA1 (CA1-s.o) at 400x magnification; scale bar = 50 *μ*m. (b–d) Mean (±SEM) change of VGLUT-1 density (normalized to respective control) on both ipsilateral (left) and contralateral (right) sides in the DG (b), CA3 (c), and CA1 (d) in two-week and 12-week postnoise-exposure animals. Both sides are pooled for comparison between groups (2w, ENT, and ET). Compared to two-week postexposure animals (2w), 12-week postexposure animals (ENT and ET) largely recovered to or near to baseline levels. Animal exhibiting tinnitus (ET) showed similar pattern of recovery in VGLUT-1 expression to animals resistant to tinnitus in all the three subregions. ^#^For comparison to the paired controls (dashed line), *p* ≤ 0.05. ^∗^For comparison between 2w, ENT, and ET groups, *p* ≤ 0.05. ml_d_: distal region of molecular layer; ml_m_: middle region of molecular layer; ml_p_: proximal region of molecular layer; h: hilus; s.o: stratum oriens; s.l: stratum lucidum; s.r: stratum radiatum; s.r_p_: proximal region of stratum radiatum; s.r_d_: distal region of stratum radiatum; s.lm: stratum lacunosum-moleculare.

In the DG (Figure 6), animals with tinnitus (ET) and animals resistant to tinnitus (ENT) exhibited similar levels of VGLUT-1 density in all four layers examined: the distal, middle, proximal regions of molecular layer, and hilus. No significant differences between ipsilateral and contralateral sides were revealed by two-way ANOVA in these four layers. In the proximal region of molecular layer, ENT animals recovered towards baseline levels compared to those examined two-week postexposure on both sides (I: *p* ≤ 0.01; C: *p* ≤ 0.001), but ET animals only showed significant recovery on contralateral side (C: *p* ≤ 0.01). Whereas ENT animals were significantly lower than the paired controls (I: *p* ≤ 0.01; C: *p* ≤ 0.01), ET animals exhibited similar levels of VGLUT-1 labeling to the baseline. Unlike other DG layers, VGLUT-1 density in the hilus was decreased relative to paired controls in both twelve-week noise exposure groups, although only ET animals showed significant reductions on both sides compared to the animals examined two-week postexposure (ET: I, *p* ≤ 0.05; C, *p* ≤ 0.05).

Like the dentate gyrus, area CA3 showed similar recovery of VGLUT-1 density regardless of tinnitus status. None of the three VGLUT-1-rich layers—stratum oriens, lucidum, and radiatum—exhibited significant differences between ENT and ET animals. Similarly, we did not see any differences between ipsilateral and contralateral sides in these layers. In stratum oriens, ENT animals showed significantly lower VGLUT-1 density than the paired controls on both sides (I, *p* ≤ 0.001; C, *p* ≤ 0.01), whereas ET animals only showed significant decreases on the ipsilateral side compared to controls (I, *p* ≤ 0.05). In stratum lucidum, chronically exposed animals did not show any changes in VGLUT-1 labeling compared the two-week time-point. But ET animals exhibited a slight, but significant, decrease in VGLUT-1 density relative to sham controls on the contralateral side (*p* ≤ 0.05). Though VGLUT-1 expression in stratum radiatum was not affected two-week postexposure, it was decreased significantly during the extended period in ENT on both sides but not in ET animals (ENT, I: *p* ≤ 0.05; C: *p* ≤ 0.01).

Similar to what we observed in DG and CA3, changes in VGLUT-1 density observed 2 weeks following noise exposure in CA1 recovered to near control levels, an effect that was evident independent of tinnitus phenotype. All four layers in CA1—stratum oriens, proximal and distal radiatum, and stratum lacunosum-moleculare—showed nearly identical changes of VGLUT-1 labeling between ENT and ET groups. The proximal region of stratum radiatum exhibited changes of VGLUT-1 density differentially between ipsilateral and contralateral sides (*p* ≤ 0.05), whereas other layers—stratum oriens, distal region of stratum radiatum, and stratum lacunosum-moleculare—did not show any differences between sides. In stratum oriens, VGLUT-1 density recovered to control levels by twelve weeks regardless of tinnitus status.

The proximal regions of stratum radiatum in both ENT and ET animals significantly recovered towards baseline levels during the extended period. The distal regions of stratum radiatum on both sides of ENT and the contralateral side of ET animals significantly recovered towards baseline levels twelve-week postexposure. Twelve weeks following the initial noise exposure, VGLUT-1 labeling in stratum lacunosum-moleculare remained lower than the baseline levels on the contralateral side of ENT and both sides of ET animals.

**Figure 7:**
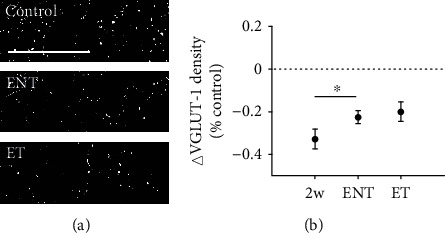
Tinnitus animals exhibit similar recovery of VGLUT-2 labeling relative to no-tinnitus animals in the granule cell layer of the dentate gyrus. (a) Representative images of VGLUT-2 labeling from granule cell layer of the dentate gyrus at 400x magnification. Scale bar = 50 *μ*m. (b) Mean (±SEM) change of VGLUT-2 density (normalized to respective control) in the DG in two-week and 12-week postnoise-exposure animals. Compared to 2-week postexposure animals (2w), 12-week postexposure animals (ENT and ET) significantly recovered towards baseline levels (only ENT significant). Animal exhibiting tinnitus (ET) showed similar pattern of recovery in VGLUT-2 expression to animals resistant to tinnitus in the granule cell layer of dentate gyrus. ^∗^*p* ≤ 0.05.

VGLUT-2 in the granule cell layer of the dentate gyrus remained lower than the paired controls in chronically exposed animals regardless of tinnitus expression (ENT: *p* ≤ 0.001; ET: *p* ≤ 0.001), though both ET and ENT animals exhibited significantly recovery of VGLUT-2 density relative to the two-week time-point (*p* ≤ 0.05) (Figure 7). Together, these results suggest that altered excitatory synapse density evident two weeks following noise exposure recovers over time, and this recovery is similar in tinnitus-vulnerable and tinnitus-resistant animals.

### 3.7. VGAT Labeling Showed Differential Recovery Depending on Tinnitus Status

Two-way ANOVA (see Table 3) reveals that VGAT expression changed equally on both sides of the brain. We thus assess expression in pooled ipsilateral and contralateral hippocampal subregions in subsequent analyses. Overall, the robust changes in hippocampal VGAT labeling observed two weeks after noise exposure largely recovered to near control levels after a 12-week recovery period. However, animals exhibiting behavioral evidence of tinnitus (ET) showed less recovery relative to animals that were resistant to tinnitus (ENT).

**Figure 8:**
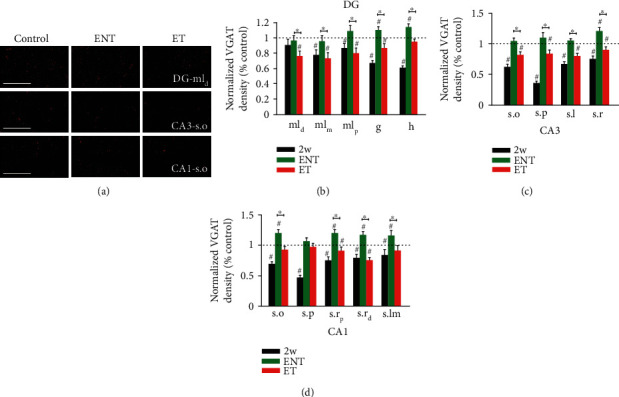
Tinnitus animals exhibit diminished recovery of VGAT labeling relative to no-tinnitus animals in the dentate gyrus (DG) and areas CA3 and CA1. (a) Representative images of VGAT labeling from distal molecular layer of the DG (DG-ml_d_), stratum oriens of CA3 (CA3-s.o), and stratum oriens of CA1 (CA1-s.o) at 400x magnification; scale bar = 50 *μ*m. (b–d) Mean (±SEM) change of VGAT density (normalized to respective control) in the DG (b), CA3 (c), and CA1 (d) in two-week and 12-week postnoise-exposure animals. Compared to 2-week postexposure animals (2w), 12-week postexposure animals (ENT and ET) largely recovered to or near to baseline levels. Tinnitus animals (ET) exhibit better recovery of VGAT labeling relative to no-tinnitus animals (ENT) in the dentate gyrus(DG) and areas CA3 and CA1. ^#^For comparison to the paired controls (dashed line), *p* ≤ 0.05. ^∗^For comparison between 2w, ENT, and ET groups, *p* ≤ 0.05. ml_d_: distal region of molecular layer; ml_m_: middle region of molecular layer; ml_p_: proximal region of molecular layer; g: granule cell layer; h: hilus; s.o: stratum oriens; s.p: stratum pyramidale; s.l: stratum lucidum; s.r: stratum radiatum; s.r_p_: proximal region of stratum radiatum; s.r_d_: distal region of stratum radiatum; s.lm: stratum lacunosum-moleculare.

In the dentate gyrus (Figure 8, Table 4), ENT animals displayed significantly higher levels of VGAT density than ET animals in all layers—the distal, middle, and proximal regions of molecular layer, granule cell layer, and hilus. ET animals maintained lower levels of VGAT labeling than the paired controls in all layers—the distal, middle, and proximal regions of molecular layer and granule cell layer—but the ENT animals recovered completely to baseline levels in the distal, middle, and proximal regions of molecular layer and exhibited significantly higher levels of VGAT density than the paired controls in the granule cell layer and hilus.

Like the dentate gyrus, ENT animals displayed greater recovery of VGAT density than ET animals in all layers of area CA3: stratum oriens, pyramidale, lucidum, and radiatum. ET animals exhibited significantly lower levels of VGAT density than paired controls in all these. ENT animals recovered to levels similar to the baseline in stratum oriens, pyramidale, and lucidum and to levels higher than the baseline in stratum radiatum.

Similar patterns of recovery were seen in area CA1, in which VGAT expression in ET animals remained diminished whereas that in ENT animals recovered completely. Except for stratum pyramidale, the other four layers—stratum oriens, proximal and distal region of radiatum, and stratum lacunosum-moleculare—demonstrated higher levels of VGAT labeling in ENT animals than in ET animals. ENT animals completely recovered above the baseline in all layers. VGAT expression ET animals remained diminished in in the proximal and distal regions of stratum radiatum. Expression in both ENT and ET animals recovered completely to the baseline in stratum pyramidale.

Taken together, our results demonstrate that VGAT expression in several hippocampal regions recovers differentially in animals that are resistant to tinnitus and animals that are vulnerable to noise-induced tinnitus. These results thus suggest a relationship between incomplete recovery of noise-induced reduction in GABAergic innervation in the hippocampus and increased susceptibility to developing tinnitus.

## 4. Discussion

In this study, we investigated the effects of noise exposure on hippocampal VGAT and VGLUT expression and determined the relevance of these effects to tinnitus. Two weeks following noise exposure, VGLUT-1 density primarily increased in the three hippocampal subregions (dentate gyrus, CA3, and CA1), but especially in CA1. VGLUT-2, which mainly localizes in the granule cell layer of the dentate gyrus, decreased by over 30%. VGAT, which is crucial for GABAergic transmission, decreased robustly in all three hippocampal subregions. Twelve weeks following the initial noise exposure, animals exhibiting tinnitus and animals exposed to noise but without tinnitus expression showed equivalent recovery in VGLUT-1 and VGLUT-2 expression, but divergent recovery in VGAT expression, in which only animals without tinnitus recovered substantially towards control levels. These findings thus demonstrate a relationship between changes in GABAergic innervation of the hippocampus and the development of tinnitus.

### 4.1. Altered Excitation-Inhibition Balance Two Weeks after Noise Exposure

VGLUTs are essential for transporting glutamate into synaptic vesicles. Three VGLUT isoforms have been identified in mammalian brains, among which VGLUT-1 and VGLUT-2 are the most abundant. VGLUT-1 and VGLUT-2 exhibit complementary distributions in the brain, both spatially and temporally. VGLUT-1 increases gradually after birth and eventually predominates over the other isoforms in telencephalic regions, including the hippocampus [51]. VGLUT-2 is expressed at high levels shortly after birth, declines with age in multiple regions [51], and is localized primarily in the thalamus and lower brainstem regions of adult animals [31, 52]. Whereas VGLUT-1 is mainly found at synapses known to show low probability of transmitter release, and VGLUT-2 is predominantly found at synapses with higher release probability in sensory pathways [53–55]. Hippocampal VGLUT-1 is synthesized by the pyramidal neurons and granule cells [52, 56, 57] or delivered by the terminals from the entorhinal cortex. VGLUT-2 terminals in the granule cell layer of the dentate gyrus arise from the mossy cells in the hilus [58] or supramammillary nucleus [54, 58]. The expression of VGLUT-2 during neural development is pivotal to the proper development of mature pyramidal neuronal architecture and plasticity [32].

Two weeks after noise exposure, VGLUT-1 expression was primarily increased in hippocampal subregions, whereas VGLUT-2 expression in the granule cell layer was decreased. These acute changes in glutamatergic transmission after moderate noise exposure are consistent with previous studies in which NMDAR 2B subunit and glutamic acid levels in the hippocampus were shown to be regulated by auditory stimulation [43, 59, 60]. Increased VGLUT-1 density two weeks after noise exposure could be due to either neural sprouting (more terminals) or terminal enlargement (each terminal contains more vesicles) and likely reflects enhanced efficacy of glutamate transmission [30]. VGLUT-2 appears to be expressed at synapses with a high release probability and VGLUT-1 at synapses with lower probabilities of release [57, 61]. The downregulation of VGLUT-2 puncta density is possibly a homeostatic adaptation for more widespread changes in VGLUT-1, to compensate for overall glutamatergic release probability [62] but could also reflect different intrinsic propensities of noise-induced changes at VGLUT-1 and -2 synapses. Future studies are needed to probe functional changes in glutamatergic transmission in the hippocampus that accompany noise exposure.

As an acute response to noise exposure, VGAT-positive puncta density changed in the opposite direction of VGLUT-1 in the hippocampus. A dramatic decrease in VGAT expression was detected two weeks following the moderate noise exposure, consistent with previous studies [43]. GABAergic inputs to hippocampus regulate the overall level of excitability in the network and contribute to hippocampal oscillations [35]. Dysfunction of GABAergic inhibition is related to several neurological diseases including epilepsy [36–38], schizophrenia [39, 40], and autism [41, 42]. The downregulation of VGAT expression levels and upregulation of VGLUT-1 levels in the hippocampus two-week postexposure reflects altered excitation-inhibition balance, which is a feature underlying the circuit dysfunction observed in various neurodevelopmental and neuropsychiatric disorders [63, 64].

Another interesting finding in this study is that no differences were seen between ipsilateral and contralateral sides of hippocampal VGLUT-2 and VGAT labeling in all layers despite the unilateral nature of noise exposure. This bilateral feature contrasts to the findings in cochlear nucleus, where noise-exposed animals show tinnitus-associated interaural asymmetry of VGLUT-1 and VGLUT-2 puncta density [62]. The cochlear nucleus is the first central stage in auditory sensory pathway, receiving signals from the ipsilateral cochlea via auditory nerve fibers. Unlike the cochlear nucleus, the hippocampus is far removed from the site of noise-damage and receives complex inputs from multiple regions, including the entorhinal cortex [26, 65], the medial septum/diagonal band of Broca (MSDB). Massive information exchange between both sides of the brain, which occurs before input to the hippocampus, might underlie the nearly identical changes in excitatory and inhibitory synapse density observed in hippocampi on both sides of the brain.

There are massive anatomical connections indirectly linking the hippocampus to sensory cortices including entorhinal cortex (EC), through which auditory and other sensory information enter the hippocampus [66, 67]. Auditory information from the cochlear nucleus is conveyed via the caudal pontine reticular nucleus, pontine central gray, and medial septum, to entorhinal cortex [68]. Input to the auditory cortex is provided by a projection from hippocampal CA1 [69].

Changes in hippocampal synaptic connectivity after noise exposure might be related to increased release of stress hormones [43, 70–72] and hence the comorbidity of tinnitus with anxiety [5] and depression [7]. The parvalbumin-positive GABAergic interneurons in the hippocampus represent an especially vulnerable population of neurons in chronic stress, which might be of key importance in the development of mood disorders [73]. The changes of VGAT and VGLUT-1 expression levels in the hippocampus two-week postexposure observed in this study are similar to what has been reported in stress animal models [74].

### 4.2. Hippocampal GABAergic but Not Glutamatergic Innervation Is Related to Tinnitus Susceptibility

To investigate whether the changes seen two-week postexposure were relevant to tinnitus, another cohort of animals was exposed to the same noise twice for tinnitus induction and followed for twelve weeks to allow tinnitus development. To make sure the GPIAS measurements reflect tinnitus rather than hearing loss, we carefully chose our noise-exposure parameters to induce only temporary threshold shifts. Consistent with this goal, ABR wave I amplitude-intensity functions indicated no suprathreshold deficits for both the ET and ENT animals at the time of tinnitus assessment with GPIAS. Twelve-week postexposure, noise-exposed animals exhibited recovery towards control levels in VGLUT-1 and VGLUT-2 density, regardless of tinnitus expression. This finding suggests that while glutamatergic innervation of the hippocampus exhibits pronounced changes following moderate noise exposure, chronic adaptations in excitatory synaptic density proceed similarly in both tinnitus-vulnerable and tinnitus-resistant animals. Acute noise-induced changes in VGAT-labeled inhibitory synapses also recovered over twelve weeks but did so differentially depending on tinnitus phenotype. VGAT labeling in animals exhibiting tinnitus remained lower but recovered to levels even higher than the controls in animals without tinnitus, suggesting a correlation between a sustained reduction in GABAergic inhibition in the hippocampus and the presence of tinnitus.

It is widely accepted that tinnitus is associated with altered neural plasticity in the central nervous system [13, 44, 75, 76]. Rauschecker et al. [77] hypothesized that if limbic structures fail to block hyperactive signals generated from auditory pathways, this noise-cancellation failure leads to chronic forms of tinnitus. In the present study, tinnitus was associated with diminished GABAergic inhibition after noise trauma, potentially resulting in increased excitability and impaired noise-cancellation function, consistent with Rauschecker's hypothesis [22]. Another hypothesis is based on the fact that some tinnitus patients can modulate their tinnitus via head, neck, and jaw contractions [78–80], which is called somatosensory tinnitus. In compensation for reduced auditory innervation after cochlear damage, somatosensory inputs to cochlear nucleus are upregulated, which is thought to be essential for initiating tinnitus and manipulating timing-dependent plasticity of cochlear nucleus fusiform cells with auditory-somatosensory stimulation alleviates tinnitus in both guinea pigs and humans [44]. The hippocampus might be involved in this process since it also responds to somatosensory stimulation via entorhinal cortex [81, 82].

## 5. Conclusions

In conclusion, this study demonstrates altered glutamatergic and GABAergic innervation in the hippocampus in noise-exposed animals. Our results demonstrate robust changes in excitatory and inhibitory synapse density in the hippocampus after noise exposure, suggesting involvement of hippocampal glutamatergic and GABAergic neurotransmission in altered auditory information processing after noise damage. After chronic tinnitus induction, VGAT but not VGLUT-1/VGLUT-2 labeling showed tinnitus-specific patterns of recovery, suggesting that rebounding inhibition in the hippocampus is potentially a protective factor against tinnitus induction.

## Figures and Tables

**Table 1:** VGAT labeling in the hippocampus was decreased significantly in most areas two weeks after noise exposure with *t*-test.

Area	Layer	*p* value	
		Ipsilateral	Contralateral
DG	Molecular layer—distal	>0.05	>0.05
	Molecular layer—middle	**≤0.01**	**≤0.05**
	Molecular layer—proximal	**≤0.01**	>0.05
	Granule cell layer	**≤0.001**	**≤0.001**
	Hilus	**≤0.001**	**≤0.001**
CA3	Oriens	**≤0.001**	**≤0.001**
	Pyramidale	**≤0.001**	**≤0.001**
	Lucidum	**≤0.001**	**≤0.001**
	Radiatum	**≤0.001**	**≤0.01**
CA1	Oriens	**≤0.001**	**≤0.001**
	Pyramidale	**≤0.001**	**≤0.001**
	Radiatum—proximal	**≤0.001**	**≤0.001**
	Radiatum—distal	**≤0.01**	**≤0.01**
	Lacunosum—moleculare	**≤0.05**	>0.05

**Table 2:** Animals with tinnitus and animals without tinnitus demonstrated similar VGLUT-1 labeling in all layers and no difference between both sides, analyzed by two-way ANOVA.

Area	Layers	*p* value for ‘tinnitus status' effect	*p* value for ‘side' effect	Interaction of tinnitus status^∗^ side
DG	Molecular layer—distal	>0.05	>0.05	>0.05
	Molecular layer—middle	>0.05	>0.05	>0.05
	Molecular layer—proximal	>0.05	>0.05	>0.05
	Hilus	>0.05	>0.05	>0.05
CA3	Oriens	>0.05	>0.05	>0.05
	Lucidum	>0.05	>0.05	>0.05
	Radiatum	>0.05	>0.05	>0.05
CA1	Oriens	>0.05	>0.05	>0.05
	Radiatum—proximal	>0.05	*≤0.05*	>0.05
	Radiatum—distal	>0.05	>0.05	>0.05
	Lacunosum—moleculare	>0.05	>0.05	>0.05

**Table 3:** Animals with tinnitus and animals without tinnitus exhibited different VGAT labeling in most layers but no difference between both sides, analyzed by two-way ANOVA.

Area	Layers	*p* value for ‘tinnitus status' effect	*p* value for ‘side' effect	Interaction of tinnitus status^∗^ side
DG	Molecular layer—distal	*≤0.05*	>0.05	>0.05
	Molecular layer—middle	*≤0.05*	>0.05	>0.05
	Molecular layer—proximal	*≤0.01*	>0.05	>0.05
	Granule cell layer	*≤0.001*	>0.05	>0.05
	Hilus	*≤0.001*	>0.05	>0.05
CA3	Oriens	*≤0.001*	>0.05	>0.05
	Pyramidale	*≤0.01*	>0.05	>0.05
	Lucidum	*≤0.001*	>0.05	>0.05
	Radiatum	*≤0.001*	>0.05	>0.05
CA1	Oriens	*≤0.001*	>0.05	>0.05
	Pyramidale	>0.05	>0.05	>0.05
	Radiatum—proximal	*≤0.001*	>0.05	>0.05
	Radiatum—distal	*≤0.001*	>0.05	>0.05
	Lacunosum—moleculare	*≤0.05*	>0.05	>0.05

**Table 4:** Animals with tinnitus and animals without tinnitus exhibited different VGAT labeling in most layers, analyzed with two-way ANOVA followed by Tukey-Kramer post hoc correction for multiple comparisons.

Area	Layers	*p* value		
		ENT vs. ET	ET vs. controls	ENT vs. controls
DG	Molecular layer—distal	**≤0.05**	≤0.001	>0.05
	Molecular layer—middle	**≤0.05**	≤0.001	>0.05
	Molecular layer—proximal	**≤0.01**	≤0.001	>0.05
	Granule cell layer	**≤0.001**	≤0.01	≤0.01
	Hilus	**≤0.001**	>0.05	≤0.001
CA3	Oriens	**≤0.001**	≤0.001	>0.05
	Pyramidale	**≤0.01**	≤0.01	>0.05
	Lucidum	**≤0.001**	≤0.001	>0.05
	Radiatum	**≤0.001**	≤0.05	≤ 0.001
CA1	Oriens	**≤0.001**	≤0.001	>0.05
	Pyramidale	>0.05	>0.05	>0.05
	Radiatum—proximal	**≤0.001**	≤0.001	≤0.05
	Radiatum—distal	**≤0.001**	≤0.001	≤0.001
	Lacunosum—moleculare	**≤0.05**	≤0.05	>0.05

## Data Availability

Data is available by request to Liqin Zhang.
